# Enhanced visible light absorption and carrier mobility in the type-II Bi_2_C_3_/GeTe van der Waals heterostructure: a first-principles study

**DOI:** 10.1039/d6ra02224c

**Published:** 2026-07-02

**Authors:** Ho Kim Dan, Huynh Thi Phuong Thuy, Le Phuong Long, Le Phuoc Dinh, Nguyen D. Hien

**Affiliations:** a Optical Materials Research Group, Science and Technology Advanced Institute, Van Lang University Ho Chi Minh City Vietnam hokimdan@vlu.edu.vn; b Faculty of Applied Technology, Van Lang School of Technology, Van Lang University Ho Chi Minh City Vietnam; c Thu Dau Mot University Ho Chi Minh City Vietnam; d Center of Scientific Research and Application, Lac Hong University No. 10 Huynh Van Nghe Str., Tran Bien Ward Dong Nai Province Vietnam phuonglong@lhu.edu.vn; e Faculty of Electricity, Electronics and Material Technology, University of Sciences, Hue University Hue City Vietnam; f Institute of Research and Development, Duy Tan University Da Nang 550000 Vietnam nguyendinhhien2@duytan.edu.vn; g School of Engineering & Technology, Duy Tan University Da Nang 550000 Vietnam

## Abstract

Vertically stacking distinct two-dimensional (2D) materials into van der Waals (vdW) heterostructures has proven to be an effective paradigm for overcoming single-layer limitations and boosting optoelectronic capabilities. In this study, we employ first-principles calculations to systematically probe the structural, electronic, transport, and optical characteristics of the Bi_2_C_3_/GeTe heterostructure. Our findings confirm the energetic stability of this heterosystem, with weak vdW forces governing the interlayer coupling. Notably, the formation of the interface enhances in-plane stiffness, thereby improving mechanical robustness. Furthermore, the heterostructure displays a type-II band alignment, facilitating the spatial separation of photogenerated electron–hole pairs. Additionally, the Bi_2_C_3_/GeTe heterostructure demonstrates a reduced band gap compared to its constituent monolayers, leading to improved visible-light responsiveness. Remarkably, the optical absorption spectrum shows a significantly broadened absorption range and enhanced absorption intensity in the visible region. The maximum absorption coefficient reaches up to 3.50 × 10^5^ cm^−1^. In addition, the Bi_2_C_3_/GeTe heterostructure exhibits remarkably high carrier mobilities for both electrons and holes, indicating excellent charge transport capability. This combination of superior carrier mobility and efficient charge separation further underscores its potential for high-performance applications.

## Abstract 2

Vertically stacking distinct two-dimensional (2D) materials into van der Waals (vdW) heterostructures has proven to be an effective paradigm for overcoming single-layer limitations and boosting optoelectronic capabilities.

## Graphical abstract

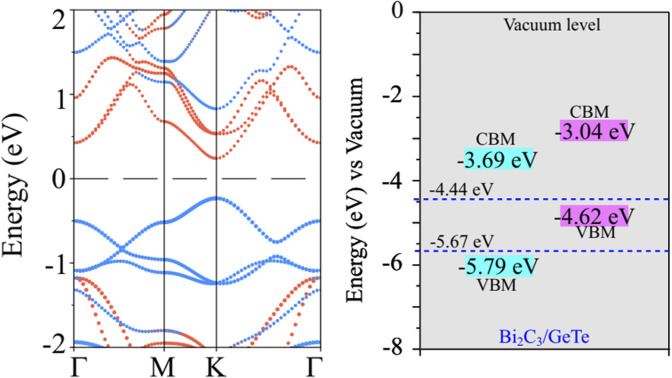

## 1 Introduction

Since the isolation of graphene,^1^ two-dimensional (2D) materials have attracted tremendous attention owing to their unique electronic, optical, and mechanical properties.^2^ Representative 2D systems, including graphene, transition metal dichalcogenides (TMDs), and black phosphorus (BP), exhibit diverse electronic characteristics ranging from gapless semimetallicity to semiconducting behavior with tunable band gaps.^3–5^ As a result, these two-dimensional structures hold immense potential for deployment in forthcoming nanoelectronic and optoelectronic technologies. However, individual 2D materials still possess intrinsic limitations. For example, graphene lacks an intrinsic band gap, TMDs generally suffer from relatively low carrier mobility, and BP exhibits poor environmental stability.^4,6,7^

Recently, the architecture of vdW heterostructures offers a powerful and flexible route toward overcoming the intrinsic performance limits found in single-layer 2D systems.^2,8,9^ By vertically stacking two different 2D semiconductors, 2D semiconductor–semiconductor (2D S–S) heterostructures can be formed, which often exhibit properties superior to those of its individual components. The main advantage of such S–S heterostructure lie in their tunable band alignment at the heterointerface. In particular, type-II (staggered) band alignment facilitates the spatial separation of photogenerated electrons and holes across different layers. Additionally, the absence of dangling bonds at the vdW interface helps suppress Fermi-level pinning effects. Motivated by these advantages, numerous high-performance vdWH systems have been investigated. For instance, the MoS_2_/WSe_2_ heterostructure has been extensively studied for its ultrafast charge transfer^10^ and robust type-II band alignment. Furthermore, the band alignment in MoSe_2_/WS_2_ heterostructures can be tuned between type-I and type-II under external stimuli,^11^ offering a versatile platform for multi-functional logic devices. Consequently, a wide variety of type-II vdW heterostructures have been theoretically designed and experimentally explored for advanced nanoelectronic and optoelectronic applications.^12–15^

Recently, Bi_2_C_3_ has been identified as a new representative of the group-XV carbide family, drawing increasing interest owing to its remarkable structural stability and distinctive electronic characteristics.^16^ Notably, both theoretical predictions and recent experimental progress have demonstrated that Bi_2_C_3_ possesses high energetic stability, and its synthesis in low-dimensional forms has recently been reported.^17^ In addition, among the group-V carbide monolayers, Bi_2_C_3_ is particularly attractive for optoelectronic applications because it possesses a direct band gap and high carrier mobility, in contrast to As_2_C_3_ and Sb_2_C_3_, which exhibit indirect band gaps.^16^ In general, direct-band-gap semiconductors are more favorable for optoelectronic and photovoltaic devices owing to their stronger light absorption and more efficient radiative electron–hole recombination. From a structural perspective, Bi_2_C_3_ features a characteristic honeycomb lattice, which gives rise to a moderate band gap along with high carrier mobility. These properties highlight its strong potential for applications in high-speed nanoscale electronic devices. Meanwhile, the germanium telluride (GeTe) monolayer, belonging to the IV–VI compound family, exhibits a hexagonal crystal structure similar to that of the GeSe monolayer, which has been successfully fabricated on a SiO_2_/Si substrate through mechanical exfoliation combined with controlled laser-thinning techniques.^18,19^ This experimental success suggests that the preparation of GeTe monolayers *via* exfoliation is highly feasible. Moreover, previous theoretical investigations have indicated that GeTe can be readily exfoliated from its bulk counterpart owing to its relatively low cleavage energy.^20^ In addition, GeTe monolayer behaves as a semiconductor with a considerable bandgap and favorable band-edge positions, rendering it suitable for applications ranging from photocatalytic water-splitting^21^ to efficient cathode catalysis in sodium–oxygen batteries.^22^

Despite the promising properties of the individual Bi_2_C_3_ and GeTe monolayers, the electronic and interfacial characteristics of their vdW heterostructure remain largely unexplored. In particular, the band alignment mechanism, interlayer charge-transfer behavior, transport and optical properties of the Bi_2_C_3_/GeTe heterostructure have not yet been systematically investigated. A deep insight into these behaviors is crucial for benchmarking the material's capabilities in high-performance optoelectronic and nanoelectronic platforms. Therefore, executing a rigorous theoretical study is highly warranted to shed light on the interfacial physics and evaluate how the Bi_2_C_3_/GeTe heterostructure can serve next-generation technologies. In this context, this work presents a systematic first-principles investigation into a newly designed Bi_2_C_3_/GeTe vdW heterostructure. The formation of a type-II band alignment is identified, which is highly desirable for efficient charge separation. The obtained results reveal that the Bi_2_C_3_/GeTe heterostructure exhibits enhanced optoelectronic performance, highlighting its potential for applications in next-generation nanoelectronic and photovoltaic devices.

## 2 Computational methods

All calculations were conducted using first-principles methods within the framework of density functional theory (DFT), as implemented in the Quantum ESPRESSO package.^23,24^ The ion–electron interaction was treated using the projector augmented-wave (PAW) method,^25^ with PAW pseudopotentials taken from the standard Quantum ESPRESSO pseudopotential library (PSLibrary).^26^ The generalized gradient approximation (GGA) in the form of the Perdew–Burke–Ernzerhof (PBE) formulation^27,28^ was selected for treating exchange–correlation energy. A cutoff energy of 520 eV was specified for the plane-wave basis set throughout the computational framework, as depicted in Fig. S1 of the SI. The explicitly included valence configurations consisted of Bi (6s^2^6p^3^), C (2s^2^2p^2^), Ge (4s^2^4p^2^), and Te (5s^2^5p^4^). A *Γ*-centered 15 × 15 × 1 Monkhorst–Pack grid was implemented to perform calculations across the Brillouin zone. Geometry relaxation was completed once the interatomic forces and total energy variations were minimized below 0.01 eV Å^−1^ and 10^−8^ eV, respectively. Spurious interactions between adjacent periodic sheets were mitigated by applying a vacuum spacing of at least 25 Å along the out-of-plane axis, which is sufficiently large to eliminate artificial interlayer coupling and exceeds the vacuum thickness adopted in previous GeTe-based heterostructure studies.^29,30^ The vdW interactions between the Bi_2_C_3_ and GeTe layers were accounted for using the Grimme DFT-D3(BJ) correction scheme with the Becke–Johnson (BJ) damping function.^31^ In addition, the electronic band structures were recalculated using the Heyd–Scuseria–Ernzerhof (HSE06) hybrid functional^32^ to obtain more reliable band gap values. To examine the thermodynamic stability of the Bi_2_C_3_/GeTe heterostructure, *ab initio* molecular dynamics (AIMD) simulations were conducted employing the Nose–Hoover thermostat with the *NVT* ensemble to regulate the temperature. The simulations were performed over a total duration of 5 ps with an integration time step of 1 fs. The phonon spectra were calculated using the PHONOPY package based on the density-functional-perturbation theory (DFTP)^33^ with a 3 × 3 × 1 supercell and a 6 × 6 × 1 *k*-point mesh.^34^ The optical properties were calculated based on the frequency-dependent complex dielectric function within the PBE framework. A total of 112 empty conduction bands were included in the calculations to accurately describe the interband electronic transitions, and a Gaussian broadening parameter of 0.05 eV was adopted. The optical response was evaluated for the out-of-plane polarization direction.

## 3 Results and discussion

**Fig. 1:**
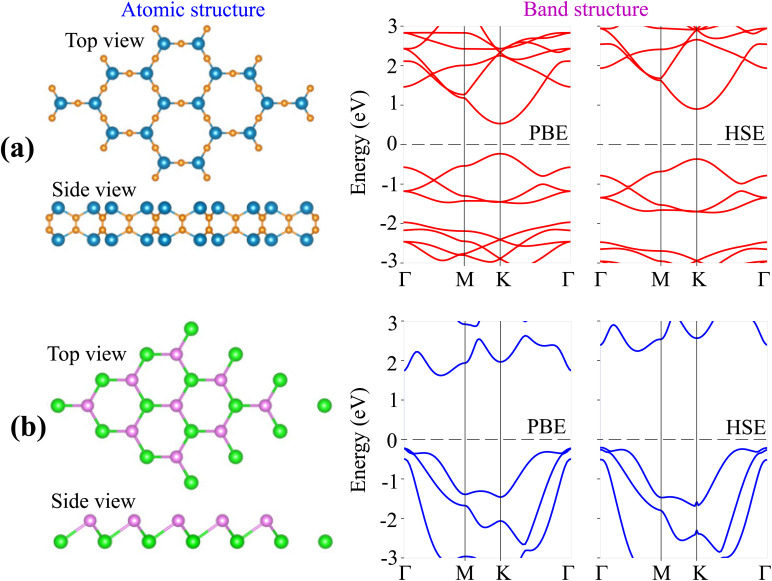
Optimized crystal geometries and electronic energy bands of pristine (a) Bi_2_C_3_ and (b) GeTe monolayers. The constituent Bi (blue), C (orange), Ge (green), and Te (purple) atoms are illustrated as colored spheres.

The optimized atomic structures and electronic band structures of the Bi_2_C_3_ and GeTe monolayers are presented in Fig. 1. The Bi_2_C_3_ monolayer exhibits a honeycomb two-dimensional atomic structure and belongs to the space group *P*6/*mmm* (no. 191). The unit cell of the Bi_2_C_3_ monolayer consists of ten atoms, in which six carbon atoms are bonded to four bismuth atoms, as displayed in Fig. 1(a). The optimized lattice parameter of the Bi_2_C_3_ is calculated to be 6.72 Å, which is highly consistent with previous theoretical reports.^35,36^ According to the band structures computed *via* both PBE and HSE functionals, the pristine Bi_2_C_3_ monolayer exhibits semiconducting behavior characterized by a direct band gap. Notably, this direct electronic transition is governed by the co-location of the valence band maximum (VBM) and conduction band minimum (CBM) at the high-symmetry *K* point of the Brillouin zone. Similarly, the GeTe monolayer adopts a buckled structure, which is similar to those of arsenene and blue phosphorene. The optimized geometry reveals strong covalent interactions between Ge and Te atoms. The calculated lattice parameter for the GeTe monolayer stands at 3.98 Å, which matches well with earlier theoretical literature.^37,38^ This material also exhibits an indirect band gap, with its VBM at the *Γ* point and CBM along the *Γ*–*M* direction in the Brillouin zone.

**Fig. 2:**
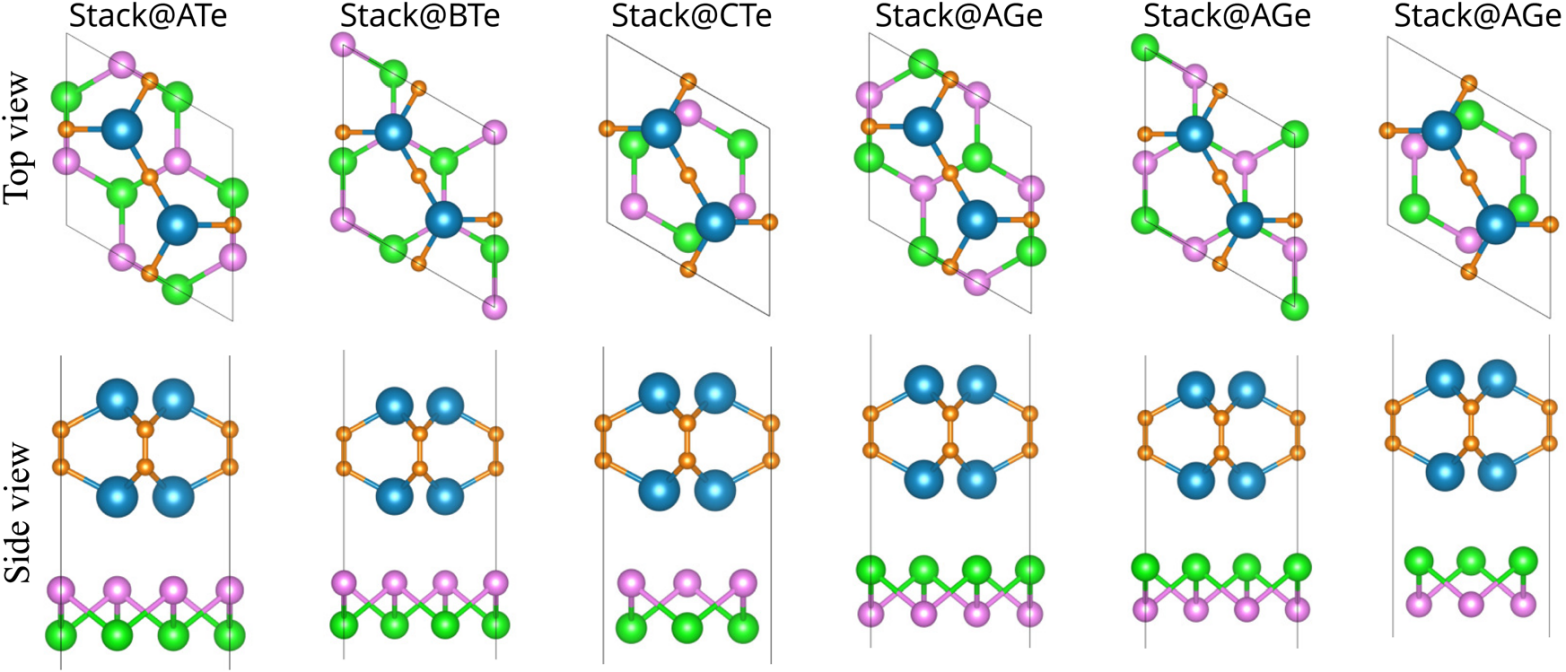
Diverse stacking configurations of the Bi_2_C_3_/GeTe heterostructure featuring both Te- and Ge-terminated surfaces, showing their optimized atomic geometries.

We now design the Bi_2_C_3_/GeTe vdW heterostructure by vertically stacking the Bi_2_C_3_ and GeTe monolayers. To reduce lattice strain during interface fabrication, we constructed the heterostructure by combining a 
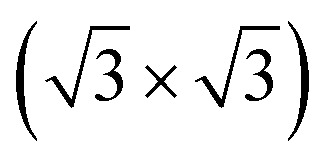
 supercell of the GeTe substrate with a (1 × 1) unit cell of the Bi_2_C_3_ monolayer. The resulting equilibrium lattice constant of the relaxed Bi_2_C_3_/GeTe vdW heterosystem is found to be 6.80 Å, which represents the mean value of the lattice parameters of both individual layers. To accommodate the lattice matching in the optimized heterostructure, the GeTe monolayer is slightly compressed, whereas the Bi_2_C_3_ monolayer is moderately stretched. The lattice mismatch between the Bi_2_C_3_ and GeTe monolayers is calculated to be only 1.3%, indicating that the formation of the Bi_2_C_3_/GeTe vdW heterostructure is structurally feasible. We consider two distinct surface terminations for the GeTe layer, namely the Te-terminated and Ge-terminated surfaces. For each termination, three high-symmetry stacking configurations are investigated, denoted as Stack@ATe(Ge), Stack@BTe(Ge), and Stack@CTe(Ge), as illustrated in Fig. 2. These configurations correspond to different relative lateral alignments between the two layers. In the Stack@ATe(Ge) configuration, the Bi atoms of the Bi_2_C_3_ layer are positioned above the centers of the Ge–Te hexagonal rings. In the Stack@BTe(Ge) configuration, the Bi atoms are located directly above the surface Te (Ge) atoms of the GeTe layer. In the Stack@CTe(Ge) configuration, the Bi atoms are situated above the Ge–Te bond sites.

After full structural relaxation, the interlayer spacing and binding energy of all stacking configurations are compared to identify the energetically favorable structure, as listed in Table 1. The relaxed interlayer separations *d* fall within the range of 3.47–3.68 Å, which clearly confirms that weak vdW forces govern the coupling between the Bi_2_C_3_ and GeTe components. These equilibrium spacings match the characteristic values of typical vdW assemblies, implying that the native electronic structures of the constituent monolayers remain predominantly intact upon interface formation. Furthermore, the Bi_2_C_3_/GeTe heterosystems featuring a Te-terminated interface display marginally smaller interlayer gaps compared to their Ge-terminated counterparts. This difference can be attributed to the higher electronegativity of Te atoms compared with Ge atoms, which leads to a relatively stronger interlayer interaction. Moreover, among the considered configurations, the Stack@BTe possesses the shortest interlayer distance, suggesting a stronger interlayer coupling in this stacking arrangement.

**Table 1:** Calculated interlayer distance, binding energy per area, band gap and band alignment of the Bi_2_C_3_/GeTe heterostructure for different stacking configurations with Te-terminated and Ge-terminated surfaces

Stacks	*d*, Å	*E*_b_, meV Å^−2^	*E*_g_, eV	Band alignment
Stack@ATe	3.52	−11.32	0.62	Type-II
Stack@BTe	3.47	−14.44	0.66	Type-II
Stack@CTe	3.51	−11.34	0.62	Type-II
Stack@AGe	3.68	−10.25	0.53	Type-II
Stack@BGe	3.62	−10.89	0.50	Type-II
Stack@CGe	3.67	−10.34	0.53	Type-II

To assess the structural stability of the Bi_2_C_3_/GeTe heterostructure, the binding energy per area (*E*_b_) is evaluated as follows:

1 *E*_b_ = [*E*_Bi_2_C_3_/GeTe_ − *E*_Bi_2_C_3__ − *E*_GeTe_]/*A*,

Here, *E*_Bi_2_C_3_/GeTe_ signifies the total energy of the optimized heterostructure. Meanwhile, *E*_Bi_2_C_3__ and *E*_GeTe_ are the respective energies computed for the isolated Bi_2_C_3_ and GeTe single layers. *A* represents the surface area of the heterostructure supercell. It is worth noting that the binding energy typically incorporates both the vdW interaction and the structural deformation potential energy caused by lattice mismatch. In the Bi_2_C_3_/GeTe heterostructure, owing to the small lattice mismatch of only 1.3% between the constituent monolayers, the energy penalty associated with lattice strain is practically negligible. Therefore, the exfoliation energy (or work of separation) can be reasonably considered equivalent to the magnitude of the calculated *E*_b_. The calculated *E*_b_ for all stacking configurations are listed in Table 1. It is found that all configurations exhibit negative *E*_b_, indicating that the formation of the Bi_2_C_3_/GeTe heterostructure is energetically favorable. In addition, the value of *E*_b_ of the Bi_2_C_3_/GeTe heterostructure is comparable with that observed in other typical vdW materials, such as graphite,^39^ BSe/As_2_C_3_ (ref. 40) and C_3_N_4_,^41^ further confirming the presence of the weak vdW interactions between Bi_2_C_3_ and GeTe layers. Among the considered stacking patterns, the Stack@BTe configuration possesses the lowest *E*_b_, indicating that it is the most energetically favorable arrangement. This minimal binding energy, combined with an equilibrium interlayer spacing of roughly ∼3.5 Å, provides evidence that weak vdW forces dictate the coupling between the Bi_2_C_3_ and GeTe layers. Such characteristics are commonly observed in vdW heterostructures and are beneficial for preserving electronic properties of Bi_2_C_3_ and GeTe monolayers.

**Fig. 3:**
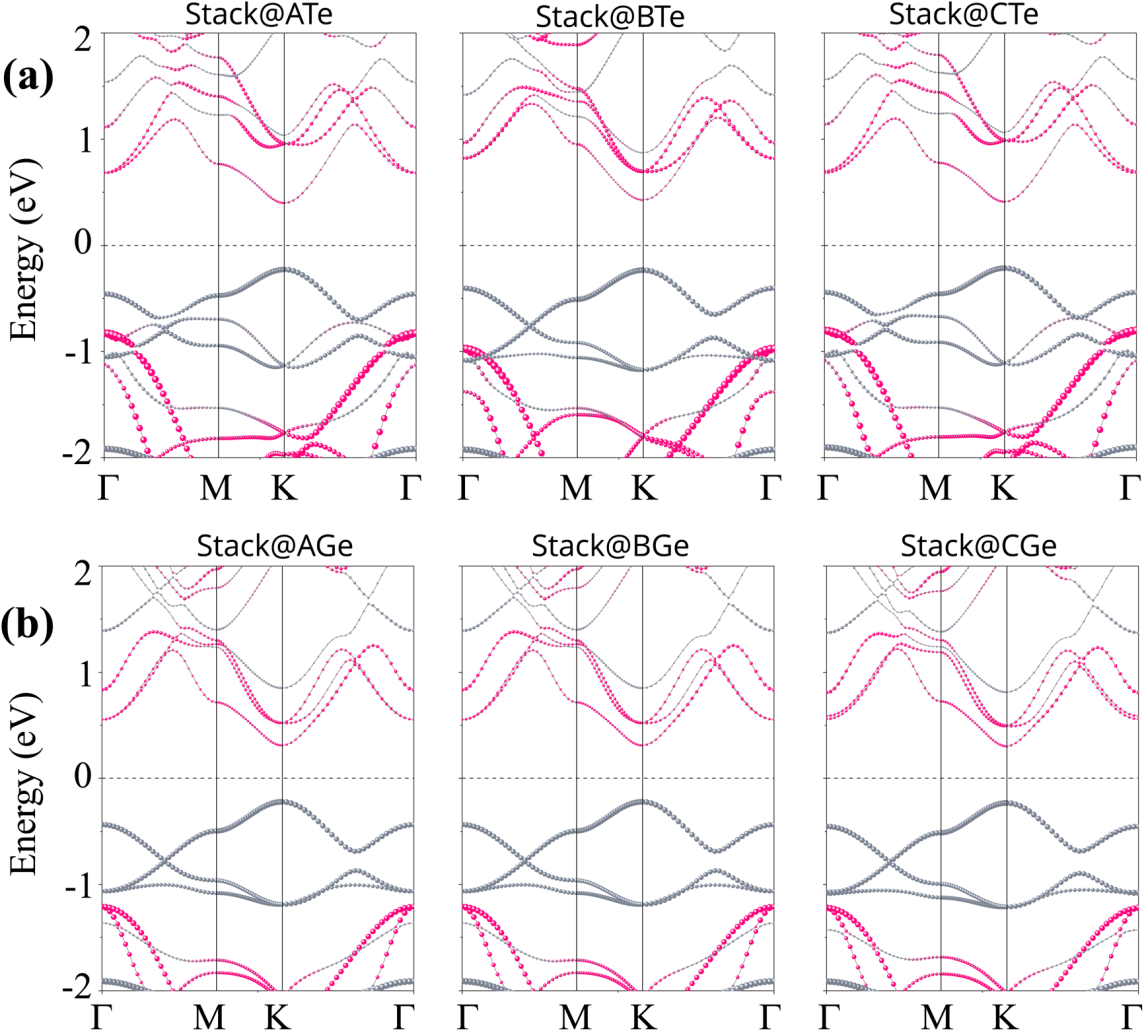
Projected band structures of Bi_2_C_3_/GeTe heterostructure with (a) Te-terminated and (b) Ge-terminated surfaces. Purple and gray lines represent the contributions of GeTe and Bi_2_C_3_ layers, respectively. The Fermi level is set to be zero and indicated by the black dashed lines.

Furthermore, we investigate the electronic properties of the Bi_2_C_3_/GeTe heterostructure for both Te-terminated and Ge-terminated surfaces, as illustrated in Fig. 3. The Bi_2_C_3_/GeTe heterostructure exhibits semiconducting behavior for both configurations. The band gaps are summarized in Table 1. Compared with those of the constituent monolayers, these values are reduced, indicating a band gap narrowing effect induced by interlayer interaction. This reduction in band gap is expected to enhance the optical absorption coefficient, thereby improving the light-harvesting capability of the heterostructure. From the projected band structures, it is evident that the VBM and CBM originate from different constituent layers. In particular, the GeTe layer primarily contributes to the CBM, whereas the VBM is governed by the Bi_2_C_3_ layer. This characteristic signifies a typical type-II (staggered) band alignment within the Bi_2_C_3_/GeTe heterostructure. Such a configuration facilitates the spatial redistribution of photo-excited carriers, leading to electron accumulation in the GeTe region and hole localization in the Bi_2_C_3_ part. This effective charge separation is highly beneficial for optoelectronic applications, particularly in photodetectors and photovoltaic devices.

**Fig. 4:**
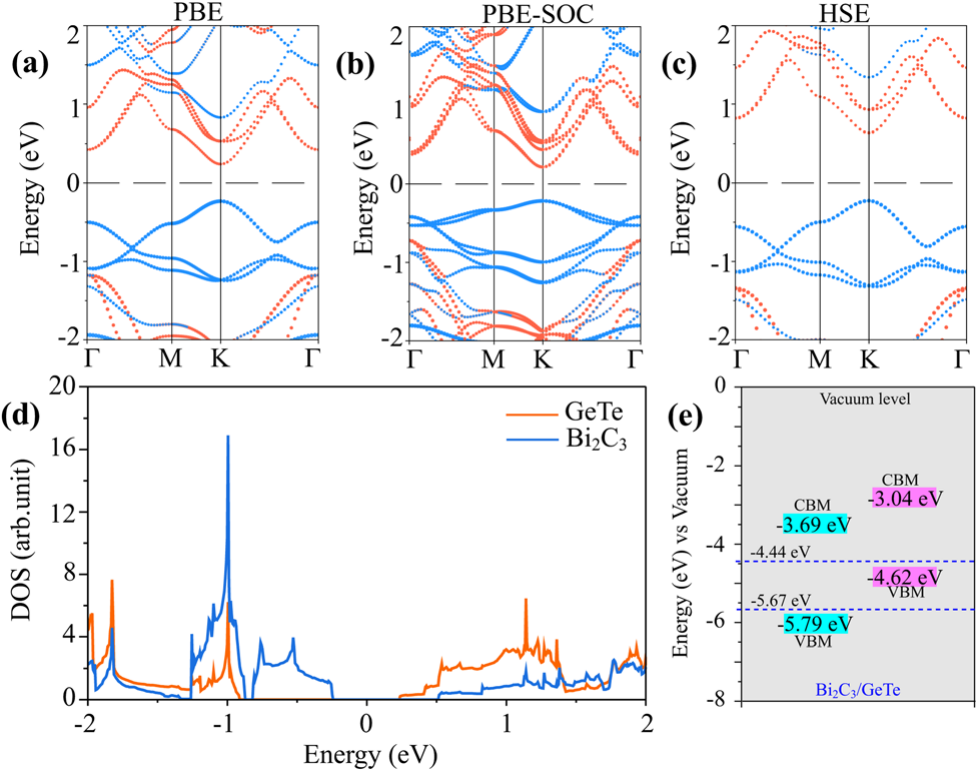
Projected band structures of Bi_2_C_3_/GeTe heterostructure for the most stable stacking configuration obtained by (a) PBE, (b) PBE + SOC and (c) HSE functional. (d) PDOS of GeTe and Bi_2_C_3_ layers in the heterostructure. (e) Vacuum-level band alignment of the Bi_2_C_3_/GeTe heterostructure.

To obtain a more reliable description of the electronic properties of the Bi_2_C_3_/GeTe heterostructure, the band structures of the most stable stacking configuration were further recalculated using the PBE + SOC and hybrid HSE06 approaches. It is well known that the conventional PBE functional generally underestimates the band gap of semiconductors. Moreover, because the heterostructure contains heavy elements such as Bi and Te, SOC may noticeably influence the electronic structure. Therefore, the inclusion of SOC is essential for a more rigorous evaluation of the band dispersion and band alignment. Fig. 4(a–c) presents the projected band structures obtained using the PBE, PBE + SOC, and HSE06 methods. The inclusion of SOC slightly reduces the band gap from 0.62 eV (PBE) to 0.58 eV (PBE + SOC), indicating a moderate effect in the heterostructure. Nevertheless, the type-II band alignment remains preserved after including SOC, demonstrating the robustness of the staggered band-edge configuration. In contrast, the HSE06 functional yields a noticeably larger band gap due to its improved treatment of exchange interactions. Despite this quantitative correction, both the band dispersion characteristics and the type-II alignment remain unchanged, with the VBM and CBM mainly contributed by the Bi_2_C_3_ and GeTe layers, respectively. Further analysis of the partial density of states (PDOS) in Fig. 4(d) reveals that p orbitals from Bi and C atoms dominate the states near the VBM, whereas Ge and Te p orbitals constitute the CBM. This agreement reinforces the staggered band nature of the Bi_2_C_3_/GeTe heterostructure. Moreover, the absence of significant hybridization near the Fermi level implies weak vdW interaction, preserving the electronic identities of each constituent monolayer. The vacuum-level band alignment of the Bi_2_C_3_/GeTe heterostructure is illustrated in Fig. 4(e). The VBM of the Bi_2_C_3_ and GeTe monolayers is located at −5.79 and −4.62 eV, respectively, while the corresponding CBM energies are −3.69 and −3.04 eV. It is clearly observed that both the CBM and VBM of the Bi_2_C_3_ layer lie at lower energies than those of the GeTe layer, demonstrating a staggered type-II band alignment. Accordingly, the valence-band and conduction-band offsets between the Bi_2_C_3_ and GeTe layers are calculated to be 1.17 and 0.65 eV, respectively.

**Fig. 5:**
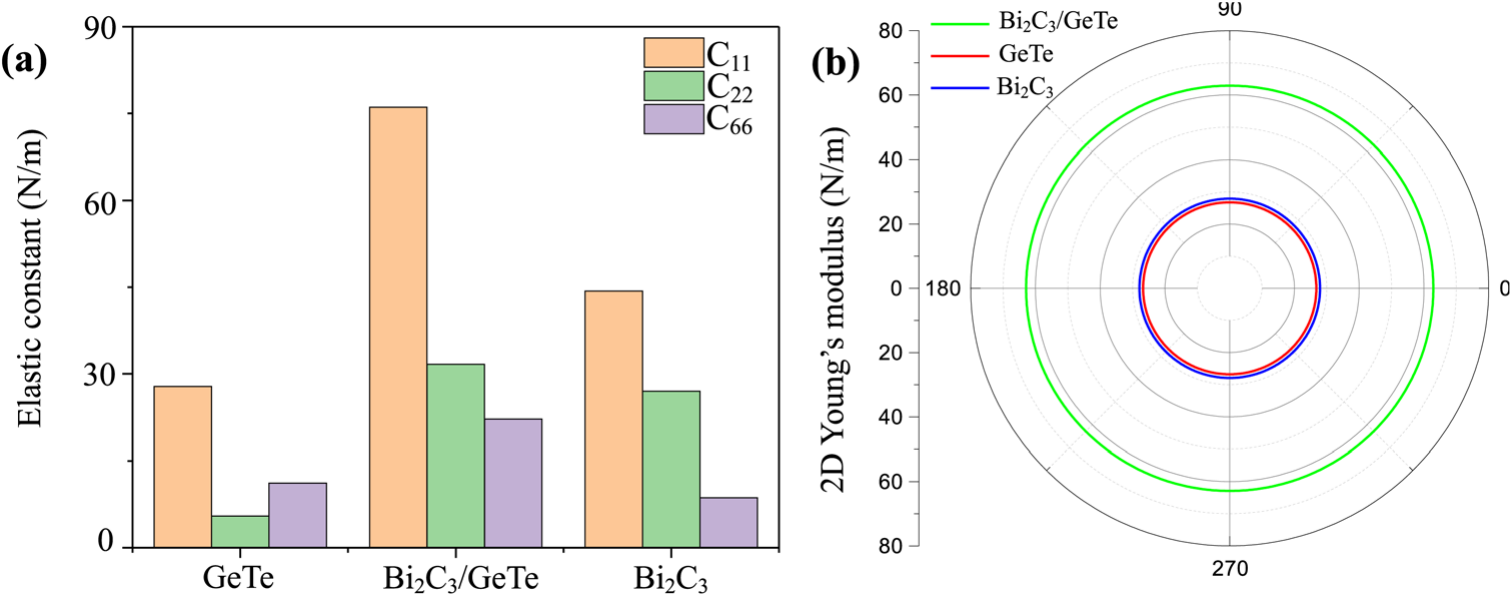
(a) Calculated elastic constants and (b) 2D Young's modulus of the Bi_2_C_3_/GeTe heterostructure and the constituent monolayers.

The mechanical robustness of the Bi_2_C_3_/GeTe heterostructure was assessed by determining its 2D Young's modulus and elastic constants, with the results presented in Fig. 5. For a two-dimensional system, the mechanical stability criteria require that *C*_11_*C*_22_ − *C*_12_^2^ > 0 and *C*_66_ > 0. The Bi_2_C_3_/GeTe heterostructure possesses reliable mechanical robustness, as its derived elastic constants meet all necessary stability indicators. Furthermore, the elastic constants of the Bi_2_C_3_/GeTe heterostructure are calculated to be *C*_11_ = 76.05 N m^−1^, *C*_12_ = 31.66 N m^−1^, and *C*_66_ = 22.20 N m^−1^. Such values are noticeably higher than those of individual Bi_2_C_3_ and GeTe films, highlighting an enhanced resistance to in-plane deformation. Furthermore, the in-plane mechanical anisotropy of the Bi_2_C_3_/GeTe heterostructure is evaluated through the orientation-dependent 2D Young's modulus, which is expressed as:^42^2
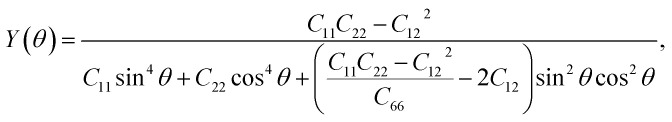


As shown in Fig. 5(b), the nearly isotropic mechanical nature is evidenced by the circular profile of *Y*(*θ*). Notably, the Young's modulus of the Bi_2_C_3_/GeTe heterostructure (62.87 N m^−1^) markedly surpasses those of standalone Bi_2_C_3_ (27.88 N m^−1^) and GeTe (26.75 N m^−1^) monolayers. Such an augmentation implies that heterostructure assembly strengthens the in-plane stiffness and structural durability. This effect originates from the interlayer vdW forces, which bolster mechanical integrity while maintaining suitable flexibility.

**Fig. 6:**
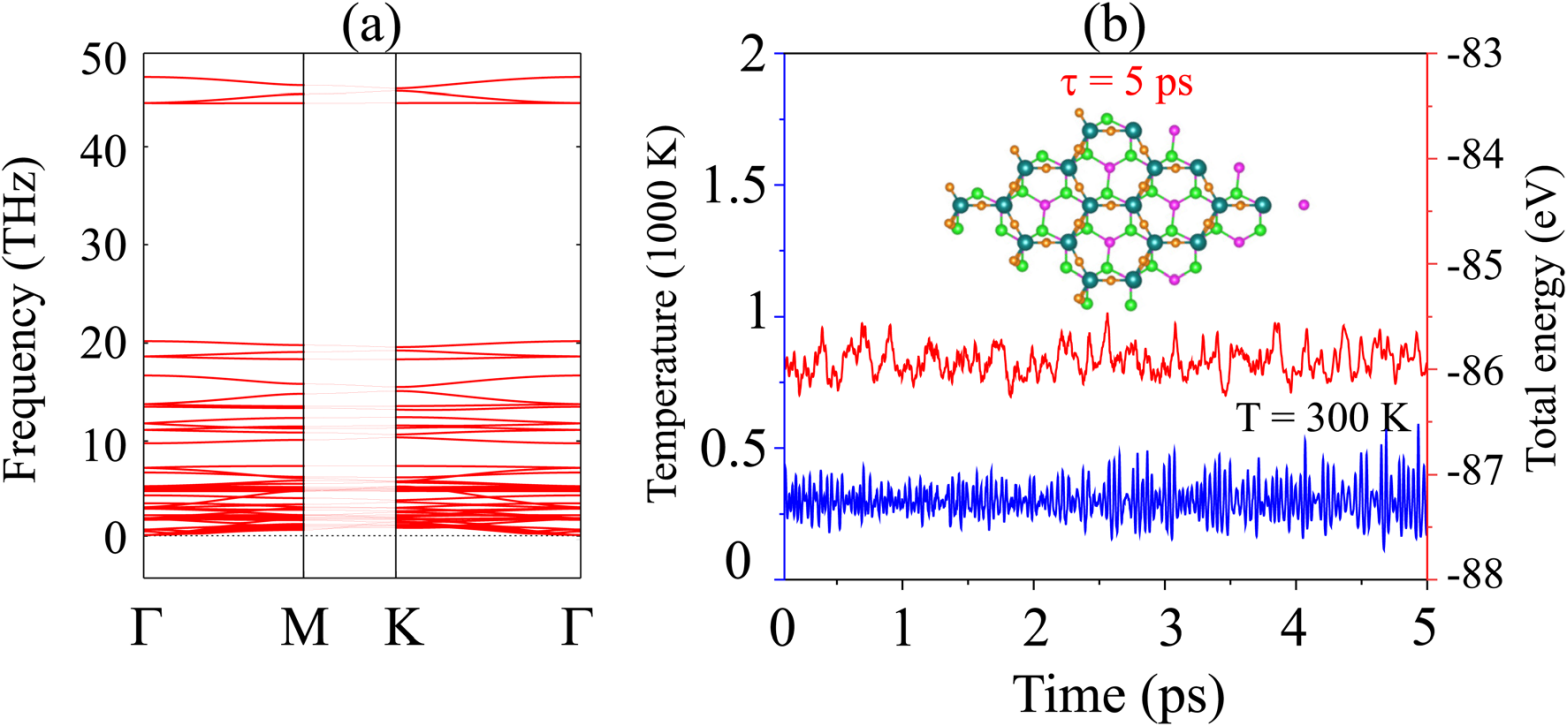
(a) Phonon spectra and (b) AIMD simulations of the temperature and total energy of the Bi_2_C_3_/GeTe heterostructure. The inset shows the final atomic configuration of the heterostructure after the AIMD simulation of 5 ps.

Additionally, the dynamical and thermal stability of the Bi_2_C_3_/GeTe heterostructure are further examined through phonon spectrum calculations and AIMD simulations. The phonon spectrum shown in Fig. 6(a) exhibits no significant imaginary frequencies throughout the entire Brillouin zone, confirming the dynamical stability of the Bi_2_C_3_/GeTe heterostructure. Furthermore, the thermal stability of the heterostructure is evaluated using AIMD simulations at room temperature. As presented in Fig. 6(b), the total energy fluctuates only slightly during the simulation without any abrupt variation, indicating that the heterostructure remains thermodynamically stable. Moreover, the final atomic configuration after the AIMD simulation retains the original structural framework without noticeable bond breaking or severe lattice reconstruction. These findings underscore that the Bi_2_C_3_/GeTe heterostructure exhibits exceptional thermal and dynamical stability, highlighting its viability for integration into practical device architectures.^15,43–45^

**Fig. 7:**
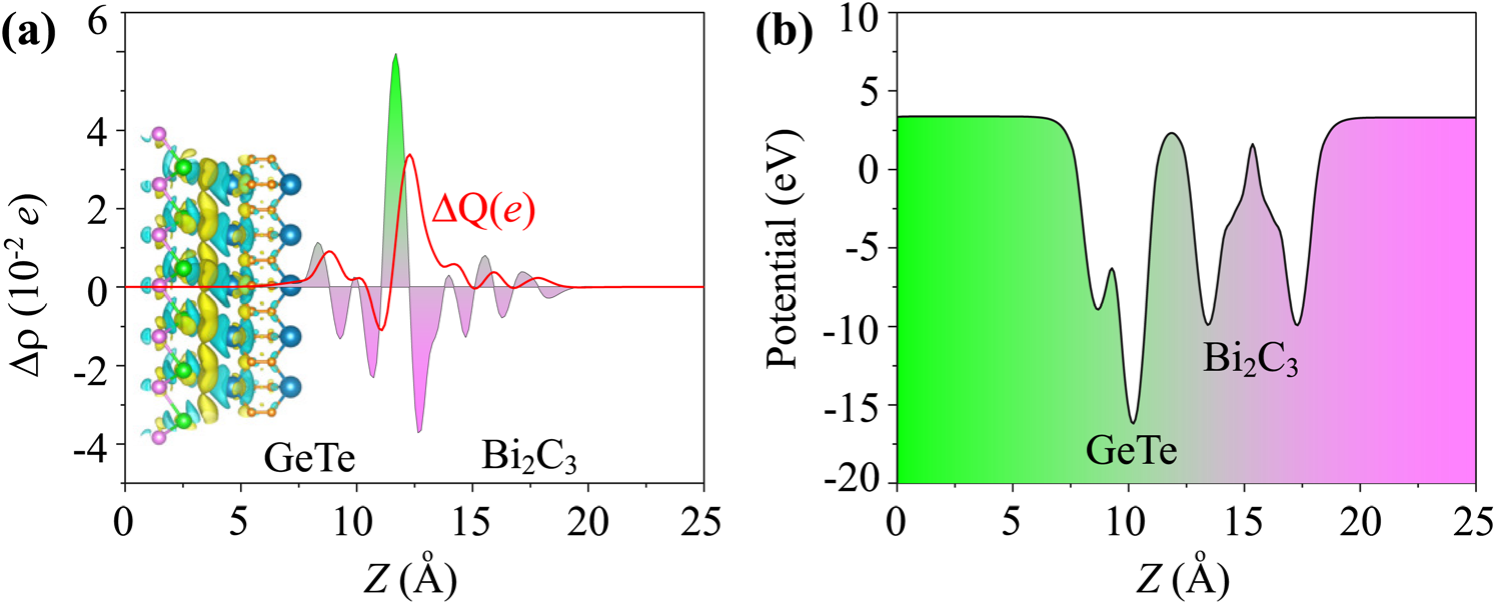
(a) Distribution of the planar-averaged charge density difference and (b) the corresponding electrostatic potential in the Bi_2_C_3_/GeTe heterostructure. Charge gain and loss are illustrated by yellow and cyan shaded areas, respectively.

To further understand the interfacial charge redistribution in the Bi_2_C_3_/GeTe heterostructure, the charge density difference is visualized and calculated, as shown in Fig. 7. The charge density difference is defined as:

3 Δ*ρ* = *ρ*_Bi_2_C_3_/GeTe_ − *ρ*_Bi_2_C_3__ − *ρ*_GeTe_

where *ρ*_Bi_2_C_3_/GeTe_, *ρ*_Bi_2_C_3__ and *ρ*_GeTe_ represent the charge densities of the Bi_2_C_3_/GeTe heterostructure, isolated Bi_2_C_3_ and GeTe monolayers, respectively. Fig. 7(a) shows that charge accumulation is mainly observed near the GeTe layer, while charge depletion appears around the Bi_2_C_3_ layer. This indicates that electrons are transferred from the Bi_2_C_3_ layer to the GeTe layer, which is consistent with the type-II band alignment discussed above. Moreover, the spatial redistribution of charge, as revealed by the charge density difference is further quantified by the cumulative charge transfer as follows:4
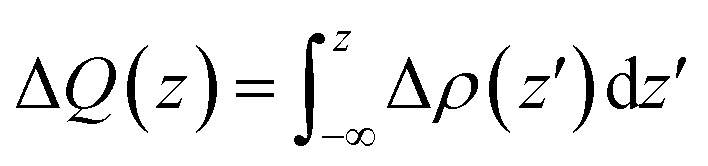


The positive values of Δ*Q*(*z*) indicate charge accumulation, while negative values correspond to charge depletion. It is clearly observed that charge accumulation occurs in the GeTe region, whereas charge depletion is present in the Bi_2_C_3_ region, confirming that electrons are transferred from Bi_2_C_3_ to GeTe. To further elucidate the interfacial electronic interaction, the electrostatic potential is analyzed, as illustrated in Fig. 7(b). It can be seen that the GeTe layer exhibits a deeper electrostatic potential than the Bi_2_C_3_ layer, indicating a pronounced potential drop across the interface. This potential difference gives rise to a built-in electric field directed from the GeTe layer toward the Bi_2_C_3_ layer. This result is in good agreement with the charge transfer behavior discussed above, where electrons migrate from Bi_2_C_3_ to GeTe. Furthermore, the work function of the Bi_2_C_3_/GeTe heterostructure is calculated to be 4.18 eV, which lies between those of the constituent monolayers, namely 4.06 eV for Bi_2_C_3_ and 5.38 eV for GeTe. This difference in work functions indicates the existence of interfacial charge redistribution upon heterostructure formation. The resulting electrostatic potential gradient not only confirms the direction of charge transfer but also plays a crucial role in promoting efficient separation of charge carriers across the interface.

**Fig. 8:**
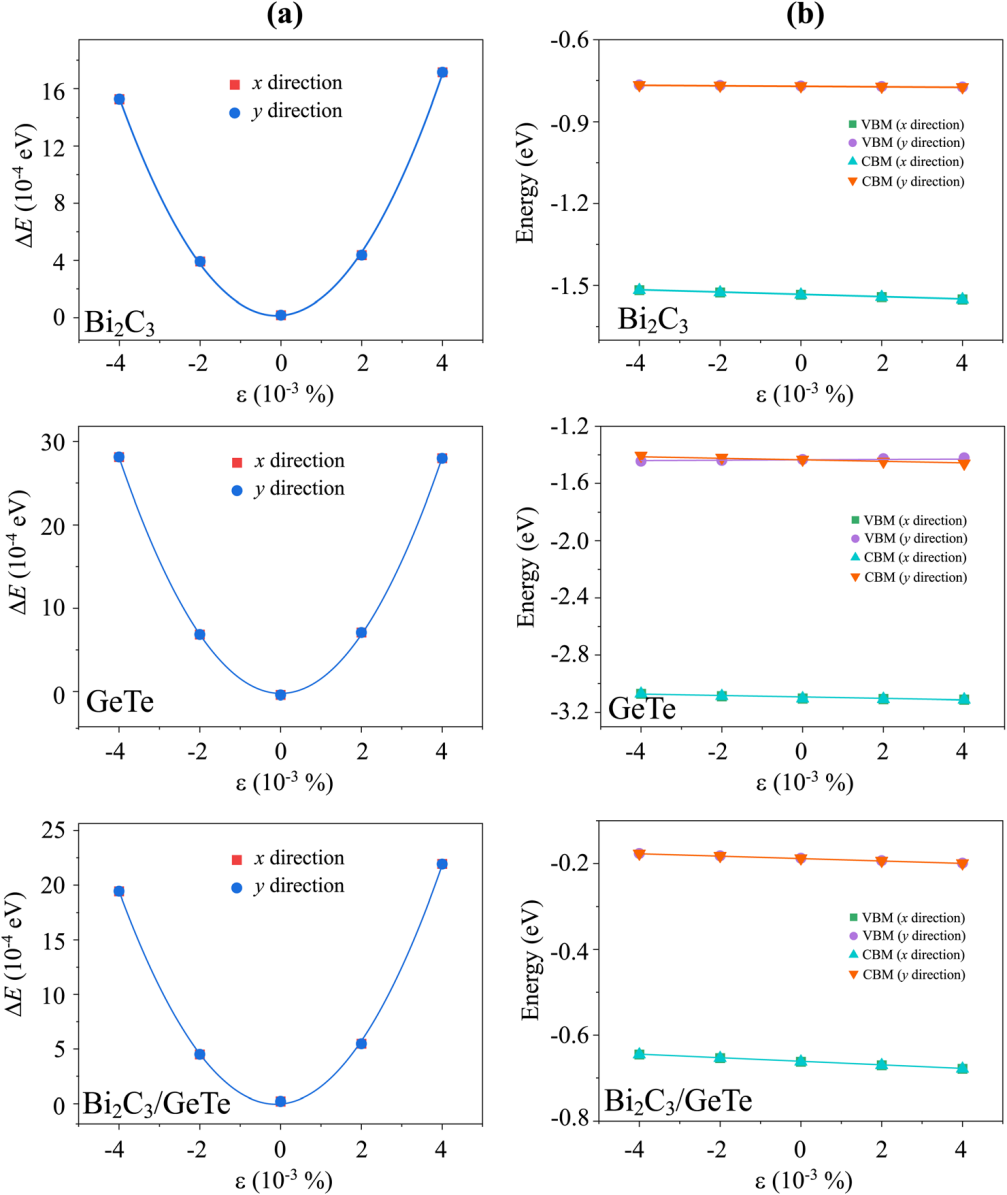
The variations in (a) total energies and (b) band edges energy of the Bi_2_C_3_, GeTe monolayers and the Bi_2_C_3_/GeTe heterostructure under small strains.

Furthermore, carrier mobility is a crucial parameter for assessing the practical applicability of a material in electronic and optoelectronic devices. Therefore, the carrier mobility of the Bi_2_C_3_/GeTe heterostructure is calculated to gain deeper insight into its charge transport properties, as expressed by:^46^5
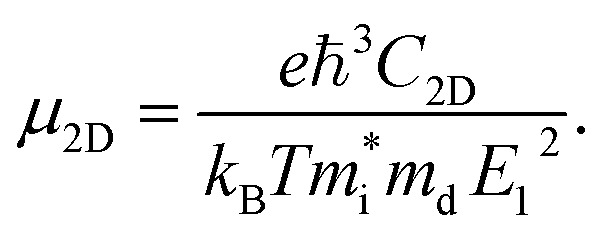
Here, *e*, *ħ*, and *k*_B_ denote the elementary charge, reduced Planck constant, and Boltzmann constant, respectively, while the temperature *T* is set to 300 K. The term *C*_2D_ = [∂^2^*E*/∂*ε*^2^]/*S* represents the in-plane elastic modulus of the two-dimensional system. The deformation potential constant *E*_1_ = ∂*E*_edge_/∂*ε* describes the shift of the CBM or VBM under small strain, as illustrated in Fig. 8. The average effective mass is defined as 
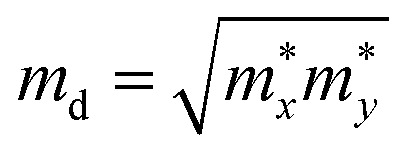
, and 
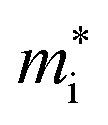
 is the carrier effective mass along the transport direction. It can be observed that the formation of the type-II Bi_2_C_3_/GeTe heterostructure leads to a noticeable reduction in the carrier effective masses compared with those of the isolated monolayers, which is beneficial for carrier transport. On the other hand, the heterostructure exhibits relatively larger deformation potential constants, as summarized in Table 2, indicating a stronger sensitivity of the band-edge energies to lattice deformation. The competition between the reduced effective masses and the increased deformation potential constants collectively determines the resulting carrier mobility of the heterostructure.

**Table 2:** Predicted transport parameters of Bi_2_C_3_, GeTe, and the Bi_2_C_3_/GeTe heterostructure along *x*/*y* directions: effective mass (m*), elastic modulus (*C*_2D_), deformation potential constant (*E*_1_), and carrier mobility (*µ*_2D_)

Systems	Transport	Carriers	*C*_2D_, N m^−1^	*E*_1_, eV	m* (*m*_e_)	*µ*_2D_, cm^2^ V^−1^ s^−1^
Bi_2_C_3_	*x*	e	84.74	−0.93	0.45	10 195
		h		−4.21	0.51	380
	*y*	e	84.74	−0.93	0.45	10 195
		h		−4.21	0.51	387
GeTe	*x*	e	42.93	1.28	0.56	1664
		h		−4.97	0.57	115
	*y*	e	42.93	1.28	0.64	1664
		h		−4.97	0.56	117
Bi_2_C_3_/GeTe	*x*	e	107.15	−2.81	0.27	3879
		h		−4.16	0.52	491
	*y*	e	107.15	−2.81	0.28	3740
		h		−4.16	0.51	500

The calculated carrier mobilities along the *x* and *y* crystallographic directions of the Bi_2_C_3_/GeTe heterostructure are summarized in Table 2. It can be observed that the electron mobility is approximately one order of magnitude higher than that of holes. Specifically, the electron mobility reaches about 4000 cm^2^ V^−1^ s^−1^, while the hole mobility is around 500 cm^2^ V^−1^ s^−1^. It is also found that the electron mobility of the Bi_2_C_3_/GeTe heterostructure is lower than that of the pristine Bi_2_C_3_ monolayer. This reduction mainly originates from the increased deformation potential constants in the heterostructure, as listed in Table 2. Since the carrier mobility is inversely proportional to the square of the deformation potential constant, the enhanced deformation potential in the heterostructure leads to a reduction in the electron mobility, despite the decrease in the carrier effective masses. In addition, the pronounced difference indicates that electron transport dominates in the Bi_2_C_3_/GeTe heterostructure. Driven by the synergistic combination of a type-II band profile and high electron mobility, this vdW heterosystem holds immense promise for next-generation optoelectronic and electronic device applications. In addition, these values of the carrier mobilities are still within the reasonable range reported for 2D-based vdW heterostructures. For example, in GeH/InSe heterostructures, the electron mobility is typically around 1400 cm^2^ V^−1^ s^−1^, while the hole mobility is much lower, approximately 90 cm^2^ V^−1^ s^−1^.^47^ Similarly, GaAs/Bi_2_Se_3_ type-II heterostructures exhibit anisotropic carrier transport with relatively moderate mobilities.^48^ In comparison, the Bi_2_C_3_/GeTe heterostructure demonstrates remarkably high carrier mobilities for both electrons and holes. This enhanced transport properties establishes the Bi_2_C_3_/GeTe heterostructure as an exceptionally viable candidate for future nanoelectronic and optoelectronic technologies.

**Fig. 9:**
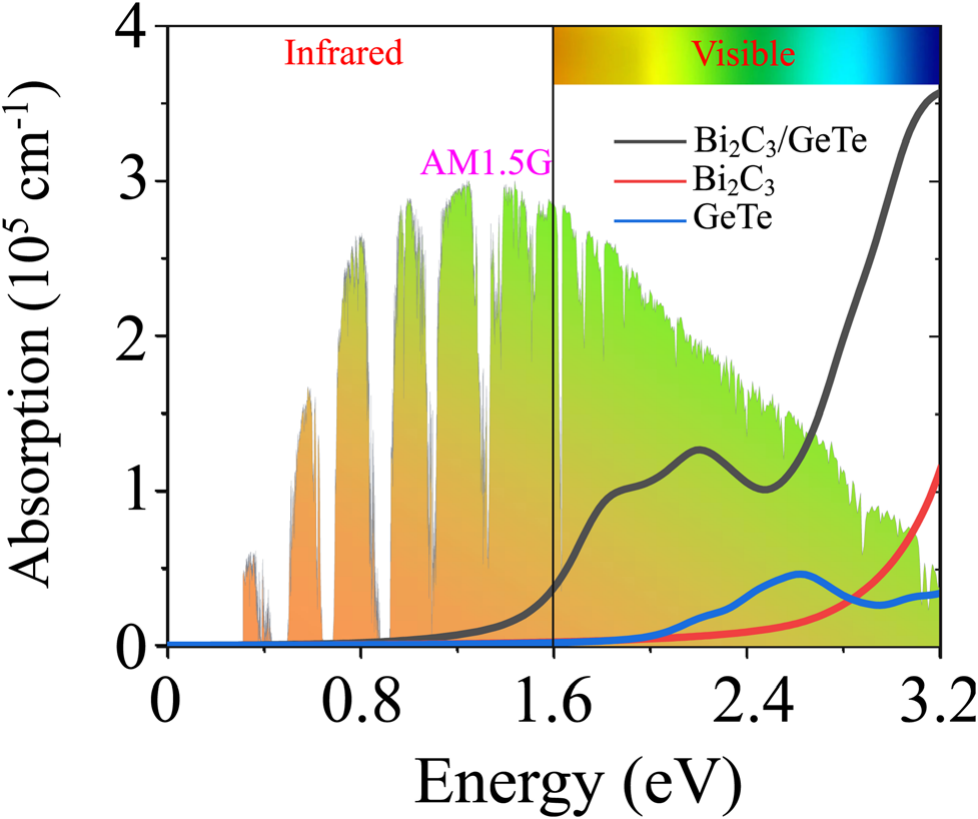
Calculated optical absorption using PBE method without SOC effect of the Bi_2_C_3_/GeTe heterostructure.

Motivated by the favorable electronic structure, including the type-II band alignment and high carrier mobility, we next examine the optical absorption properties of the Bi_2_C_3_/GeTe heterostructure to gain further insight into its potential for optoelectronic and photovoltaic applications. The optical absorption coefficient of the Bi_2_C_3_/GeTe heterostructure is defined as follows:^49^6
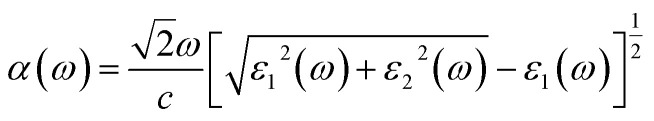
where *c* and *ω* denote the speed of light and the angular frequency of the incident photon, respectively. *ε*_1_(*ω*) and *ε*_2_(*ω*) represent the real and imaginary parts of the complex dielectric function. Furthermore, the optical absorption coefficient was calculated using the effective physical thickness of the Bi_2_C_3_/GeTe heterostructure. The optical absorption spectra of the Bi_2_C_3_/GeTe heterostructure using PBE method without SOC effect are presented in Fig. 9. It is clearly observed that, compared to the individual monolayers, the heterostructure exhibits a broader absorption range along with a significantly enhanced absorption intensity in the visible region. Notably, the maximum absorption coefficient reaches as high as *α*_max_ = 3.50 × 10^5^ cm^−1^, which is approximately three times higher than that of the Bi_2_C_3_ monolayer and nearly seventy times greater than that of the GeTe monolayer. Furthermore, this strong optical absorption is comparable to previously reported values for other two-dimensional vdW heterostructures, such as HfSe_2_/InAs,^50^ HfS_2_/AlSe^51^ and GaAs/SiH.^52^ This remarkable enhancement indicates a strong light–matter interaction within the heterostructure. Finally, although SOC can quantitatively influence the electronic band structures due to the presence of heavy Bi and Te atoms, our current optical simulations are primarily intended to provide a reliable qualitative evaluation of the light-absorption characteristics. In addition, the independent-particle approximation employed here systematically neglects many-body excitonic effects (electron–hole couplings). In low-dimensional materials, reduced dielectric screening typically leads to strong excitonic interactions, which would introduce a redshift in the absorption edge and enhance peak intensities. Nevertheless, our PBE-level independent-particle calculations successfully capture the essential physical trends and offer a computationally efficient, foundational understanding of the underlying optical transitions.

## 4 Conclusions

In summary, a comprehensive first-principles investigation of the Bi_2_C_3_/GeTe vdW heterostructure has been carried out to elucidate its fundamental properties and application potential. The heterostructure maintains structural integrity upon stacking, with interlayer interactions dominated by weak vdW forces, while exhibiting improved in-plane stiffness, indicating enhanced mechanical stability compared to the isolated monolayers. The electronic analysis confirms the formation of a type-II band alignment, which is advantageous for spatial charge separation across the interface. This feature, combined with the reduced band gap, suggests improved efficiency in light-induced charge generation and transfer processes. Moreover, the heterostructure demonstrates significantly strengthened optical absorption in the visible region, along with an extended absorption range, highlighting its suitability for solar energy harvesting. Importantly, the calculated carrier mobilities reveal that both electrons and holes can move efficiently within the heterostructure. The synergistic combination of mechanical robustness, favorable band alignment, strong light absorption, and high carrier mobility underscores the potential of the Bi_2_C_3_/GeTe heterostructure for integration into advanced nanoelectronic and optoelectronic systems. These findings provide useful theoretical insights for the rational design of high-performance vdW heterostructures.

## Conflicts of interest

There are no conflicts to declare.

## Supplementary Material

RA-016-D6RA02224C-s001

## Data Availability

The data that support the findings of this study are available from the corresponding author upon reasonable request. Supplementary information (SI) is available. See DOI: https://doi.org/10.1039/d6ra02224c.
