# Supplementary material for: Enhanced visible light absorption and carrier mobility in the type-II Bi2C3/GeTe van der Waals heterostructure: a first-principles study
Source: RSC Adv. 2026 Jul 2;16(34):32156–66. doi: 10.1039/d6ra02224c (PMC13325946; doi:10.1039/d6ra02224c)
Supplement: RA-016-D6RA02224C-s001 [file RA-016-D6RA02224C-s001.pdf]

Supplementary Information (SI) for RSC Advances.
This journal is © The Royal Society of Chemistry 2026

Supporting Information (SI)

Enhanced visible light absorption and carrier mobility in the type-II Bi_2C_3/GeTe van der Waals heterostructure: A first-principles study

Ho Kim Dan,[1,2,*] Huynh Thi Phuong Thuy,[3] Le Phuong Long,[4] Le Dinh Phuoc,[5] and Nguyen D. Hien[6,7,†]

[1]*Optical Materials Research Group, Science and Technology Advanced Institute, Van Lang University, Ho Chi Minh City, Vietnam.*

[2]*Faculty of Applied Technology, School of Technology, Van Lang University, Ho Chi Minh City, Vietnam*

[3]*Thu Dau Mot University, Ho Chi Minh City, Viet Nam*

[4]*Center of Scientific Research and Application, Lac Hong University, No. 10 Huynh Van Nghe Str, Tran Bien Ward, Dong Nai Province, Vietnam.*

[5]*Faculty of Electricity, Electronics and Material Technology, University of Sciences, Hue University, Hue, Vietnam*

[6]*Institute of Research and Development, Duy Tan University, Da Nang 550000, Vietnam*

[7]*School of Engineering & Technology, Duy Tan University, Da Nang 550000, Vietnam*

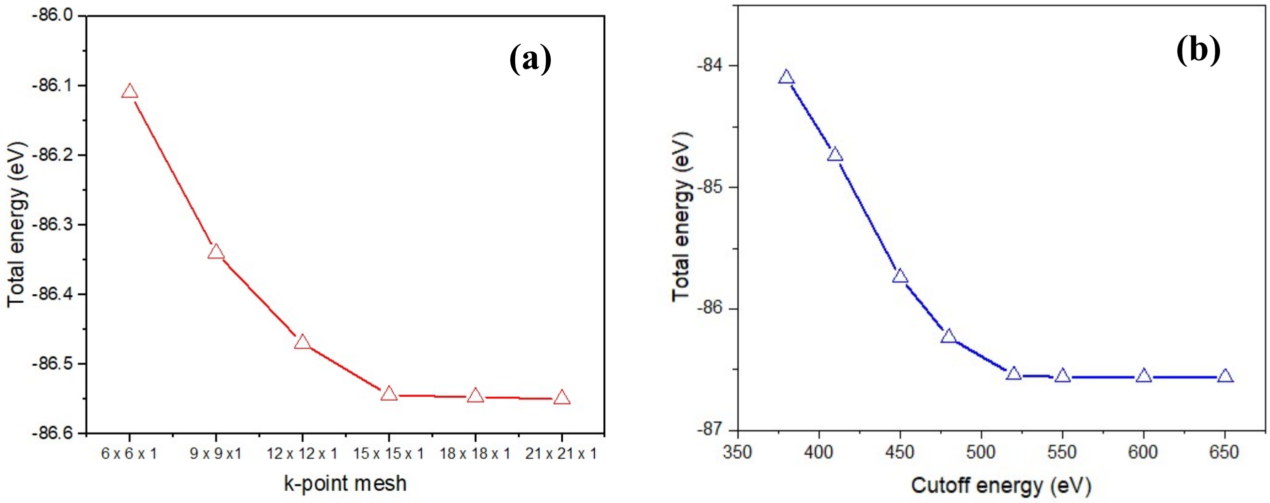

Fig. S1. The convergence tests for the (a) k-point mesh and (b) cutoff energy of the Bi2C3/GeTe heterostructure.
